# Simple analytical method using ultraviolet spectral dataset and chemometrics for the authentication of Indonesian specialty ground roasted coffee with different botanical and geographical indications

**DOI:** 10.1016/j.dib.2023.109820

**Published:** 2023-11-19

**Authors:** Diding Suhandy, Meinilwita Yulia, Agus Arip Munawar, Kusumiyati Kusumiyati

**Affiliations:** a Spectroscopy Research Group (SRG), Laboratory of Bioprocess and Postharvest Engineering, Department of Agricultural Engineering, Faculty of Agriculture, The University of Lampung, Jl. Prof. Dr. Soemantri Brojonegoro No.1, Bandar Lampung 35145, Indonesia; b Department of Agricultural Technology, Lampung State Polytechnic, Jl. Soekarno Hatta No. 10, Rajabasa Bandar Lampung 35141, Indonesia; c Department of Agricultural Engineering, Syiah Kuala University, Banda Aceh, Indonesia; d Department of Agronomy, Universitas Padjadjaran, Bandung, Indonesia

**Keywords:** Food authentication, Geographical indications, PCA, SIMCA, Specialty coffee, Spectral data, UV spectroscopy supervised classification

## Abstract

The possible application of a simple analytical method based on a UV (ultraviolet) spectral dataset coupled with SIMCA (soft independent modeling of class analogy) for authentication of Indonesian specialty ground roasted coffee with different botanical and geographical indications (GIs) was demonstrated. Three types of Indonesian specialty ground roasted coffee were used: GIs arabica coffee from Gayo Aceh (96 samples), GIs liberica coffee from Meranti-Riau (119 samples), and GIs robusta coffee from Lampung (150 samples) with 1 g weight of each sample. All samples were extracted using hot distilled water and 3 mL aqueous filtered samples were pipetted into a 10 mm quartz cell. Original UV spectral datasets were recorded in the range of 190–399 nm. The pre-processed spectral dataset was generated using three simultaneous different preprocessing techniques: moving average smoothing with 11 segments, standard normal variate (SNV), and Savitzky-Golay (SG) first derivative with window size and polynomial order value of 11 and 2. The supervised classification based on the SIMCA method was applied for preprocessed selected spectral data (250–399 nm). The PCA data showed that GIs coffee with different botanical and geographical indications can be well separated. The SIMCA classification was accepted with 100 % of correct classification (100 % CC). This dataset demonstrated the potential use of UV spectroscopy with chemometrics to perform simple and affordable authentication of Indonesian specialty ground roasted coffee with different botanical and geographical indications (GIs).

Specifications Table

**Table:** 

Subject	Food Science Agricultural and Biological Sciences
Specific subject area	UV Spectroscopy, Spectral analysis for food authentication
Data format	Raw Analyzed Preprocessed Presented as *.xls* and *.unsb* file formats
Type of data	Graph Spectroscopic data Excel data Figure
Data collection	The original ultraviolet (UV) spectral datasets of Indonesian specialty ground roasted coffee samples were acquired in aqueous solution using a benchtop UV–Vis spectrometer (Genesys™ 10S UV–Vis, Thermo Scientific, USA) in a transmittance mode. The preprocessed spectral dataset was obtained by simultaneously applying three spectral preprocessing techniques: moving average smoothing with 11 segments, standard normal variate (SNV), and Savitzky-Golay (SG) first derivative with window size and polynomial order value of 11 and 2.
Data source location	Laboratory of Bioprocess and Postharvest Engineering, Department of Agricultural Engineering, Faculty of Agriculture, The University of Lampung, Jl. Prof. Dr. Soemantri Brojonegoro No.1, Bandar Lampung, 35145, Indonesia South latitude 5°21’ and East longitude 105°14′
Data accessibility	Datasets are available in this article and can be found in Mendeley data: Repository name: Data identification number: *(or DOI or persistent identifier)* Direct URL to data: Instructions for accessing these data:

## 1 Value of the Data

• Spectral dataset acquired from UV spectroscopy provides a simple, easy, and affordable authentication method of Indonesian specialty ground roasted coffee with different botanical and geographical indications (GIs).
• UV spectral dataset can be benefited those who are working and concerned with food authentication, especially for coffee authentication, from growers, retailers, industry, and coffee shops as well as the government.
• UV spectral dataset can be employed in foods and agricultural products industries, especially for food quality evaluations purpose: authenticating and food traceability.
• The authentication of Indonesian specialty ground roasted coffee with different botanical and geographical indications is easy to be realized due to the low-cost UV spectrometer. It will help developing countries establish an authentication system based on UV spectroscopy shortly.
• The established coffee authentication system based on UV spectroscopy may lead to a certification system of Indonesian specialty ground roasted coffee. This certification is benefitable for both producers and customers and provides fair trading of specialty coffee.

## 2 Data Description

In 2007, the Indonesian government officially announced Government Regulation No. 51 about Geographical Indication (GIs). According to this regulation, GIs can be defined as any indication which identifies goods and/or a product as originating from a particular region of which its geographical environment factors including nature, labour, or a combination of both factors are attributable to a given reputation, quality, and characteristics of the produced goods and/or product [1]. In total up to April 2020, 59 Indonesian products have been approved for GIs including several Indonesian Arabica, Liberica, and Robusta coffee such as Arabica Gayo-Aceh (certificate number ID G 000 000 006), Liberica Meranti-Riau (ID G 000 000 041) and Robusta Lampung (ID G 000 000 026) [1].

**Fig. 1:**
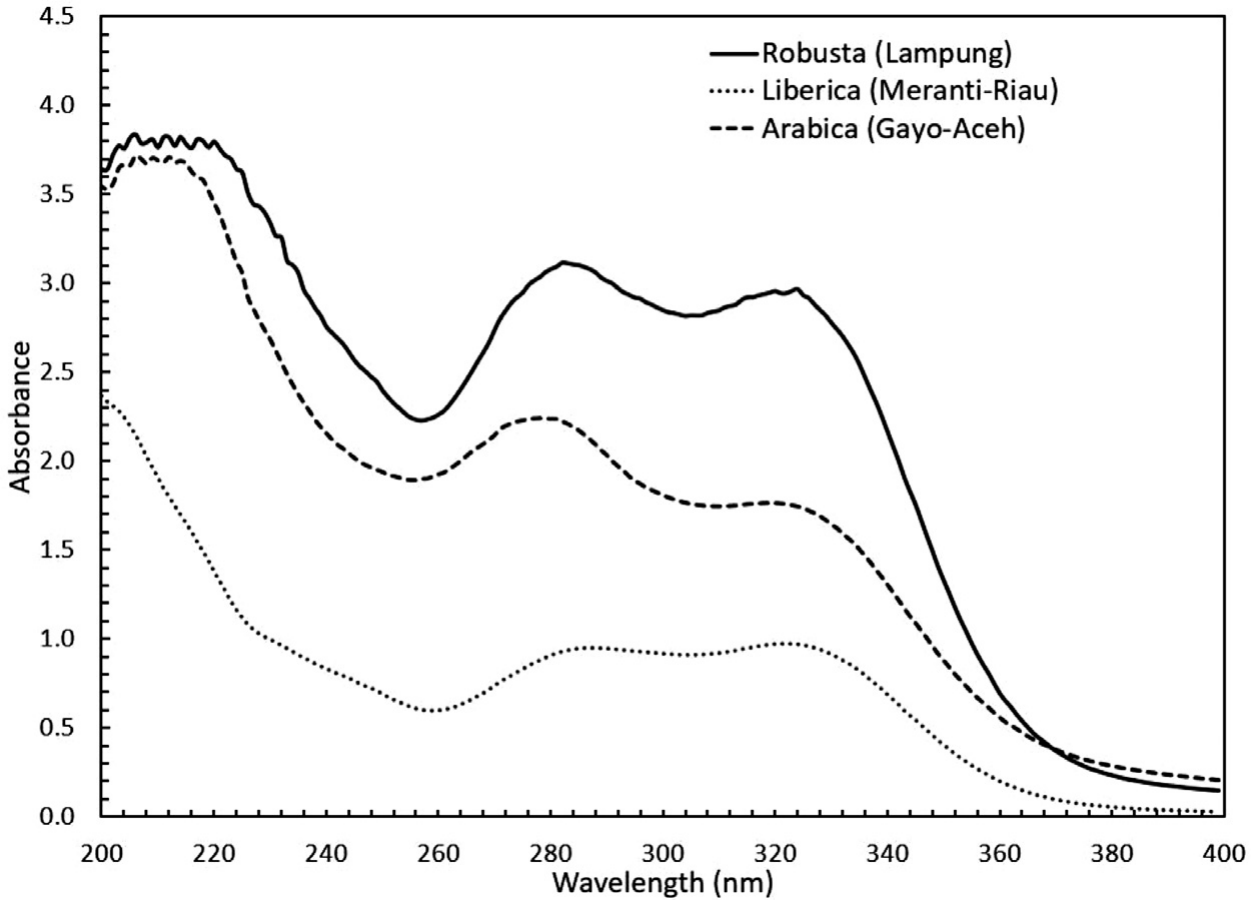
The average of the original UV spectral dataset of Indonesian specialty ground roasted coffee samples with different botanical and geographical indications (GIs) in the range of 190–399 nm.

**Fig. 2:**
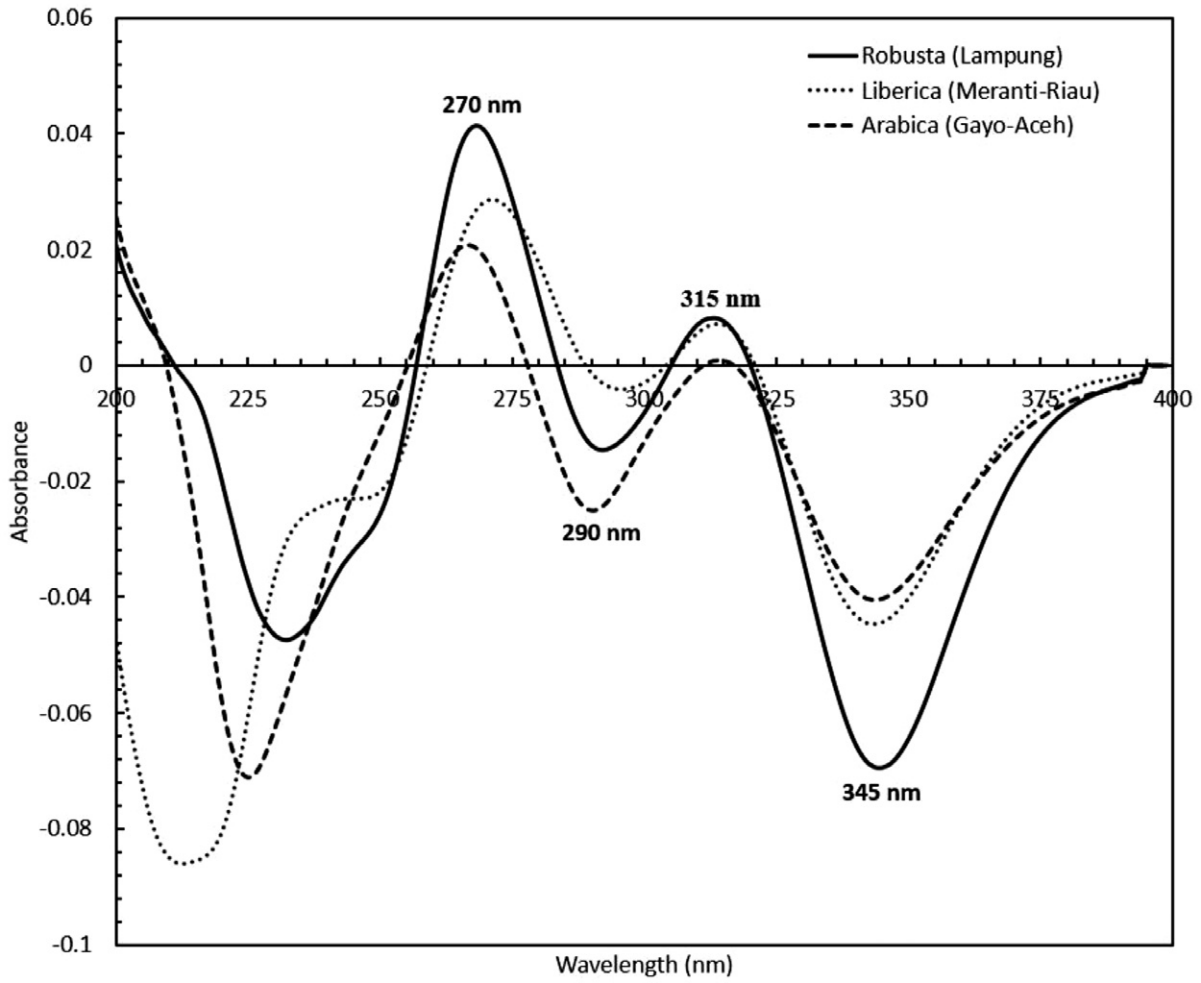
The average preprocessed UV spectral dataset of Indonesian specialty ground roasted coffee samples with different botanical and geographical indications (GIs) in the range of 190–399 nm.

Concerning the low-cost spectroscopy method, Suhandy and Yulia propose to develop an authentication for Indonesian specialty ground roasted coffee using UV spectroscopy along with the SIMCA method. The averaged original UV spectral dataset from 365 samples with different botanical and geographical indications (GIs) were presented in Fig. 1. To correct the spectral information, three different spectral preprocessing namely, moving average smoothing with 11 segments, standard normal variate (SNV), and Savitzky-Golay (SG) first derivative with window size and polynomial order value of 11 and 2 were applied simultaneously as presented in Fig. 2. Several peaks were observed at 270 nm (related to the maximum absorption of caffeine), at 290 nm and 315 nm (related to the presence of chlorogenic acids and trigonelline) [2,3]. The preprocessed spectral dataset in a selected spectral window of 250–399 nm was used for PCA (principal component analysis) calculation and developing SIMCA (soft independent modeling of class analogy) classification. The result of PCA was presented in three different plots. The first plot is Hotelling's T^2^ versus Q-residual presented in Fig. 4. This plot is useful to detect any potential outliers in the spectral dataset. Any outlier must be removed in further chemometrics calculation. The second plot is a score plot of the first and second principal components (PC1 and PC2) to map the sample separation as presented in Fig. 5. The third plot is x-loadings versus wavelength as presented in Fig. 6. This plot is important to investigate the variable or specific wavelength that is responsible for the separation of the coffee samples. SIMCA is one of the popular and widely used supervised classification methods in spectroscopy. SIMCA model for each class of Arabica Gayo, Liberica Meranti-Riau, and Robusta Lampung was developed using calibration and validation samples. The performance of each SIMCA model was evaluated using a prediction sample set. The classification results for each class were visualized using Cooman's plot as presented in Fig. 7, Fig. 8, Fig. 9. All prediction samples were properly classified to their corresponding classes resulting in 100 % correct classification for all classes.

## 3 Experimental Design, Materials and Methods

### 3.1 Sample Preparations

**Fig. 3:**
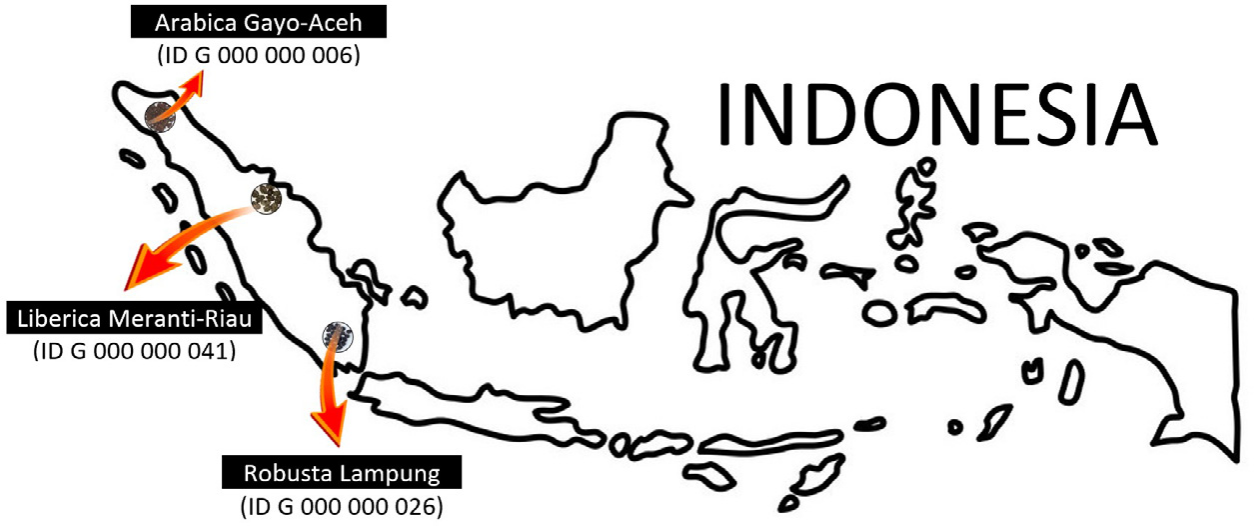
The origin of coffee samples used in the datasets from three different botanical and geographical indications (GIs).

Indonesian specialty coffee green bean samples were directly collected from trusted farmers in Indonesia from three different botanical and geographical indications (GIs): Arabica Gayo-Aceh, Liberica Meranti-Riau, and Robusta Lampung as depicted in Fig. 3. The coffee samples were subjected to medium roasting at 200°C for 10 minutes using a home coffee roaster machine and were mechanically grounded using a home coffee grinder (Sayota). Particle size in coffee powder has a great influence on the UV spectral dataset [2]. For this, all coffee samples were sieved through a nest of U. S. standard sieves (mesh number of 40) on a Meinzer II sieve shaker (CSC Scientific Company, Inc. USA) for 10 minutes to obtain a homogenous particle size of 420 µm. These experiments were performed at room temperature (around 27-29°C). Each sample weighed 1 gram and was extracted further distilled and diluted using hot distilled water [4]. For multivariate analysis, the samples were randomly divided into three sets, namely calibration (111 samples), validation (73 samples), and prediction sets (61 samples).

### 3.2 UV Spectral Dataset Acquisition

Three mL of aqueous coffee samples were pipetted in the 10 mm of quartz cell. All the UV spectral data were acquired using a dual-beam UV-Visible benchtop spectrometer (Genesys 10s UV–Vis, Thermo Scientific Inc., Madison, WI), equipped with a high-intensity xenon lamp and dual Silicon photodiodes as a detector. Spectra were measured between 190 and 399 nm with an interval of 1 nm.

### 3.3 Spectral Data Preprocessing and Chemometrics

The original UV spectral dataset of 365 samples was exported to The Unscrambler® X version 10.4 (64-bit) (Camo Software AS, Oslo, Norway) for further processing and analysis including spectral preprocessing and chemometrics calculation. The preprocessed spectral data were obtained by applying simultaneously three different preprocessing techniques, namely, moving average smoothing with 11 segments, standard normal variate (SNV), and Savitzky-Golay (SG) first derivative with window size and polynomial order value of 11 and 2. In general, smoothing was used to reduce the noise and improve the signal-to-noise ratio (SNR). SNV and derivative are frequently used spectral preprocessing methods for scatter correction, removing baseline offsets, and enhancing the resolution of overlapped peaks [5]. The chemometrics calculation including principal component analysis (PCA) and soft independent modeling of class analogy (SIMCA) was performed on the preprocessed spectral dataset using selected intervals 250-399 nm.

### 3.4 Unsupervised Classification Using PCA

**Fig. 4:**
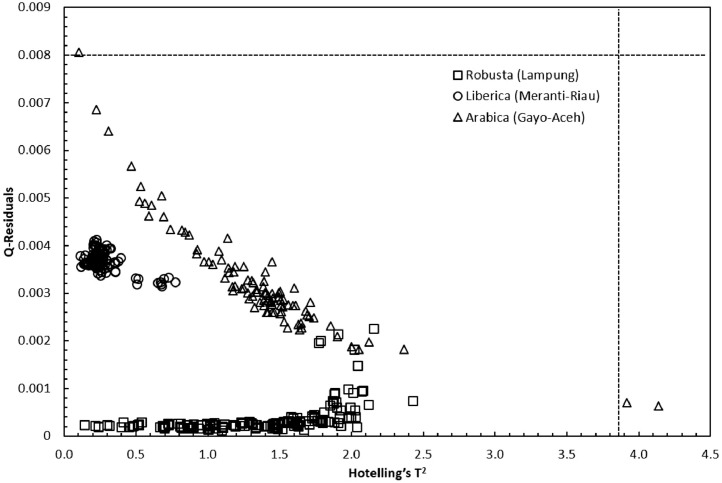
Q-residual vs. Hotelling's T^2^ statistic plot of Indonesian specialty ground roasted coffee samples with different botanical and geographical indications (GIs) calculated with a 5 % confidence level based on preprocessed spectra in the range of 250–399 nm.

**Fig. 5:**
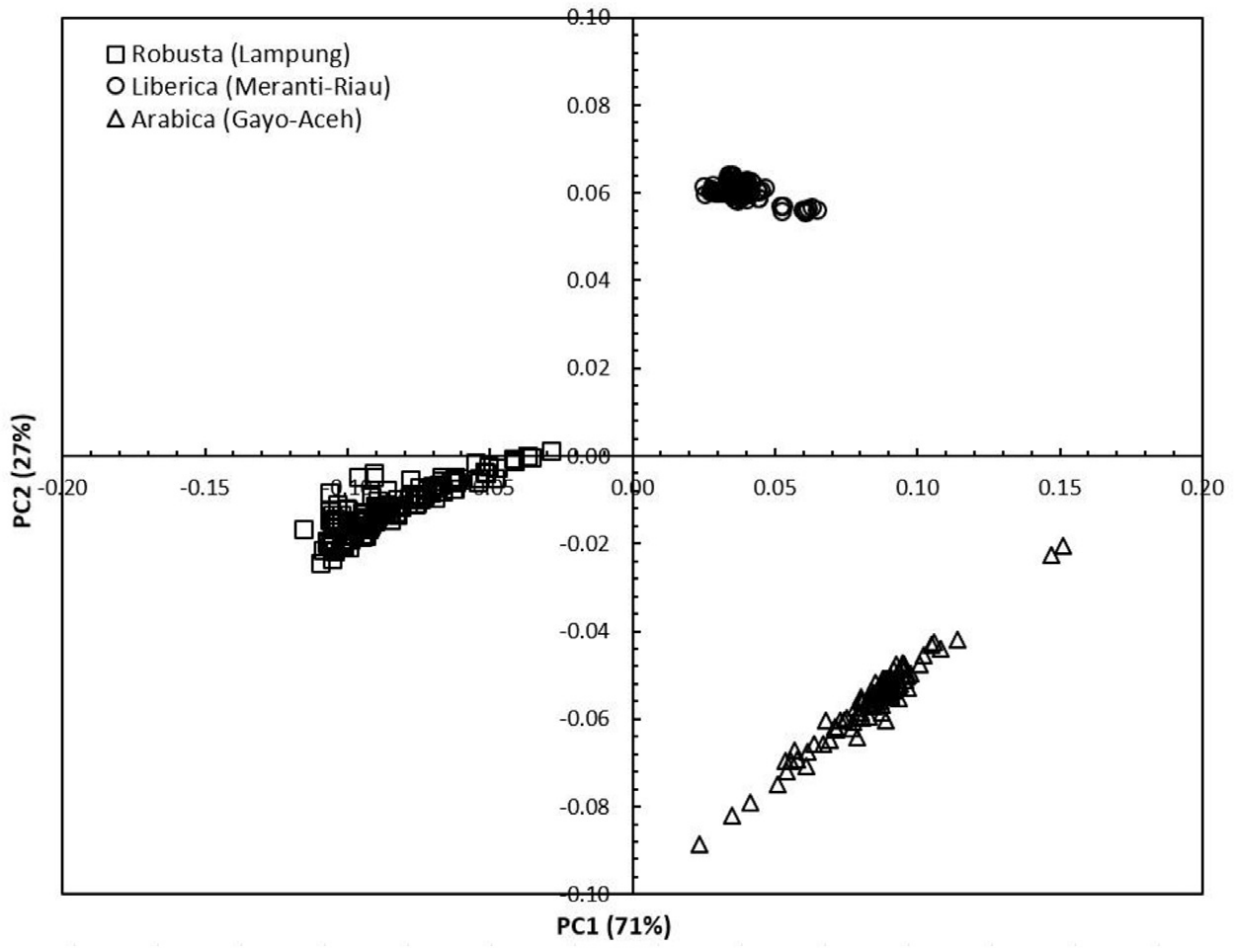
The PCA score plot of Indonesian specialty ground roasted coffee samples with different botanical and geographical indications (GIs) calculated based on preprocessed spectra in the range of 250–399 nm.

**Fig. 6:**
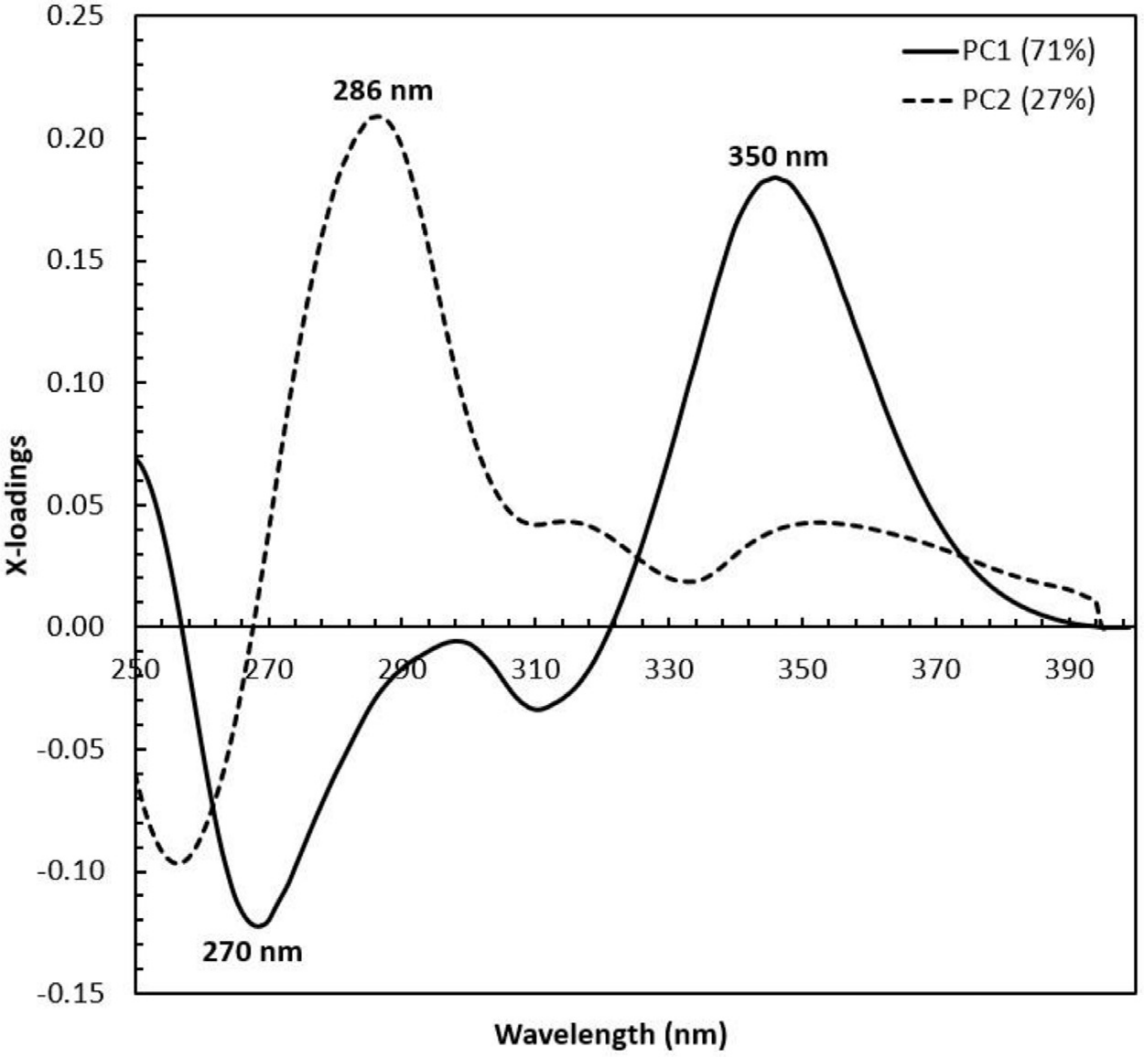
The loading plot for the first and second principal components was calculated based on preprocessed spectra in the range of 250–399 nm.

PCA was widely used for reducing the dimensionality of the spectral dataset [6]. PCA score plot was utilized to detect the possible outliers through Hotelling's T^2^ statistics and to perform unsupervised classification of Indonesian specialty ground roasted coffee samples with different botanical and geographical indications (GIs). Hotelling's T^2^ is the variation within the PCA model, while Q-residual is used to measure the dimensional data in the model [7]. For guidance, a sample was considered as an outlier assuming the Hotelling's T^2^ and Q-residual values are greater than the 95 % confidence interval or 5 % confidence level (black dotted line). Fig. 4 shows that all samples were located in the left lower part of the plot, and Hotelling's T^2^ and Q-residual values were lower than the 95 % confidence interval (black dotted line). Therefore, no outlier was detected, and this led to the use of all 365 samples for further analysis. The first two principal components (PC1 and PC2) were plotted for preprocessed spectra as depicted in Fig. 5. PC1xPC2 accumulated to 98 % of the total variance (PC1=71 % and PC2=27 %). A clear separation was observed between the three GIs, which indicated that UV spectroscopy has the potential for the classification of Indonesian specialty ground roasted coffee with different botanical and geographical indications (GIs). A plot of x-loadings versus wavelengths was presented in Fig. 6. Several important wavelengths with high x-loadings responsible for the separation of GIs coffee samples were identified at 270 nm, 286 nm, and 350 nm. Those wavelengths are closely related to the absorbance of several important chemical compounds in aqueous coffee such as caffeine, chlorogenic acid, and trigonelline [2,3].

### 3.5 Supervised Classification Using SIMCA

**Fig. 7:**
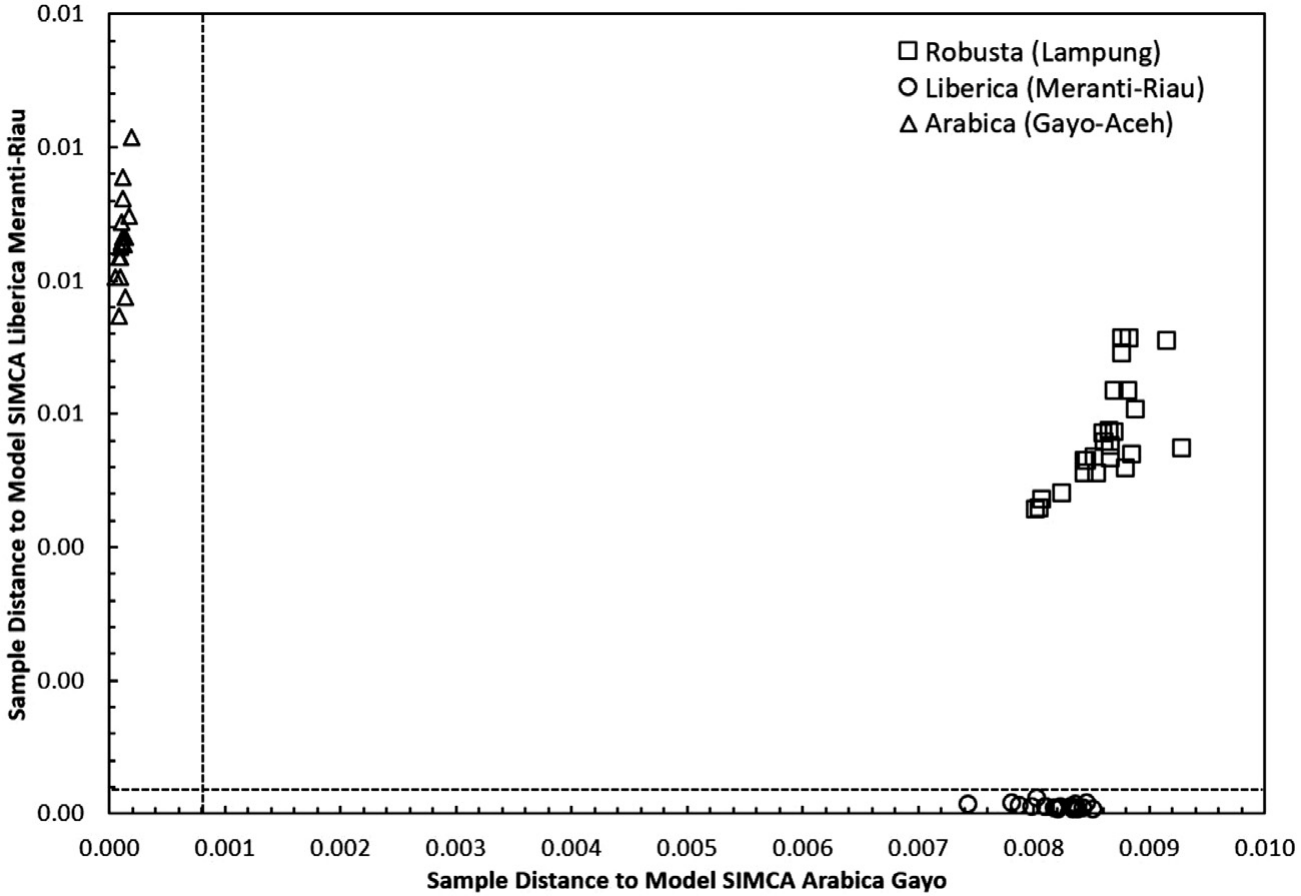
The Cooman's plot of SIMCA analysis (95 % confidence limit) using model SIMCA of Arabica Gayo and Liberica Meranti-Riau.

**Fig. 8:**
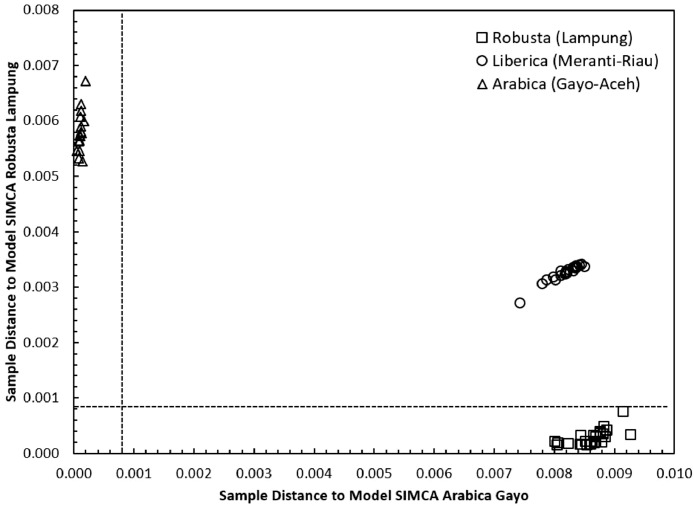
The Cooman's plot of SIMCA analysis (95 % confidence limit) using model SIMCA of Arabica Gayo and Robusta Lampung.

**Fig. 9:**
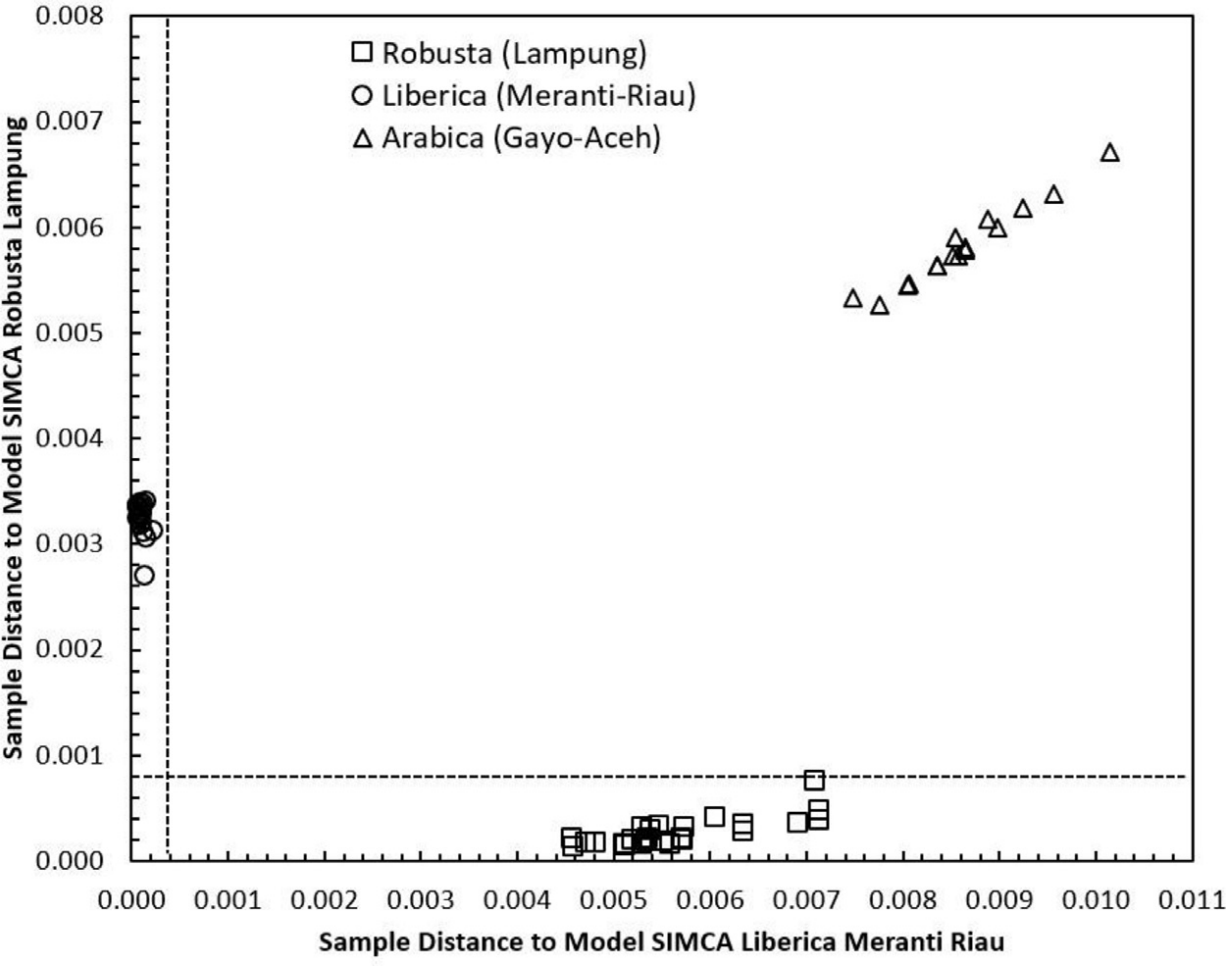
The Cooman's plot of SIMCA analysis (95 % confidence limit) using model SIMCA of Liberica Meranti Riau and Robusta Lampung.

SIMCA is one of the supervised classification methods and is frequently used in the classification of spectroscopic data [8]. SIMCA model for each class of Arabica Gayo, Liberica Meranti-Riau, and Robusta Lampung was developed using a calibration sample set using individual PCA in each class. The result of classification using SIMCA was presented using Cooman's plot. There are three pairwise classes to be tested and Cooman's plot was presented in Fig. 7, Fig. 8, Fig. 9: Arabica Gayo versus Liberica Meranti-Riau, Arabica Gayo versus Robusta Lampung, and Liberica Meranti-Riau versus Robusta Lampung. The black dashed lines in Cooman's plot is membership line with a 95 % confidence limit that divided the membership area into four quadrants. In Fig. 7, the x-axis and y-axis show the distance to Arabica Gayo and Liberica Meranti-Riau samples. All Arabica Gayo samples were plotted in the upper left quadrant and properly classified into the Arabica Gayo class (16 samples). Liberica Meranti-Riau samples were located in the right lower quadrant (20 samples), indicated to be properly classified as Liberica Meranti-Riau class. There are no samples plotted in the left lower part (belonging to both classes). Robusta Lampung (25 samples) were successfully rejected for both Arabica Gayo and Liberica Meranti-Riau class and plotted in the upper right quadrant. Similar results were observed for other pairwise classes as can be seen in Figs. 8 and 9. It showed the capability of the developed SIMCA model to classify Indonesian specialty ground roasted coffees according to their botanical and geographical origins.

## Limitations

Not applicable.

## Ethics Statement

The authors have read and follow the ethical requirements for publication in Data in Brief and confirming that the current work does not involve human subjects, animal experiments, or any data collected from social media platforms.

## CRediT authorship contribution statement

**Diding Suhandy:** Conceptualization, Methodology, Writing – review & editing, Writing – original draft. **Meinilwita Yulia:** Data curation, Software, Validation, Writing – original draft, Writing – review & editing. **Agus Arip Munawar:** Visualization, Investigation, Writing – original draft. **Kusumiyati Kusumiyati:** Software, Investigation, Supervision, Writing – review & editing.

## Data Availability

Spectral Dataset for the Authentication of Indonesian Specialty Ground Roasted Coffee (Original data) (Mendeley Data).
